# Supplementary figures and images for: Knowledge of telemedicine and its associated factors among health professional in Ethiopia: A systematic review and meta-analysis
Source: PLoS One. 2024 Apr 18;19(4):e0301044. doi: 10.1371/journal.pone.0301044 (PMC11025815; doi:10.1371/journal.pone.0301044)

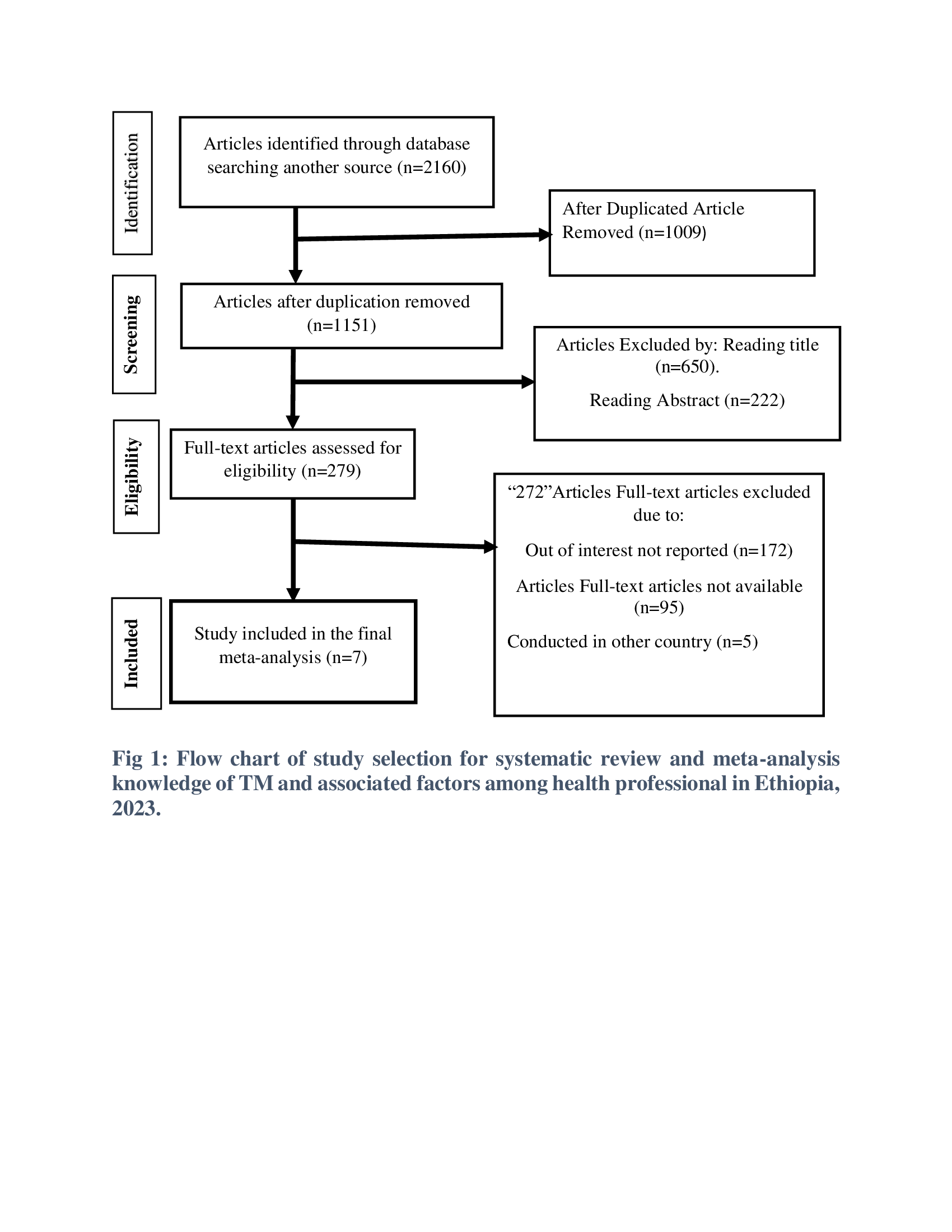

Supplement: S1 Fig — (TIFF) [file pone.0301044.s002.tiff]

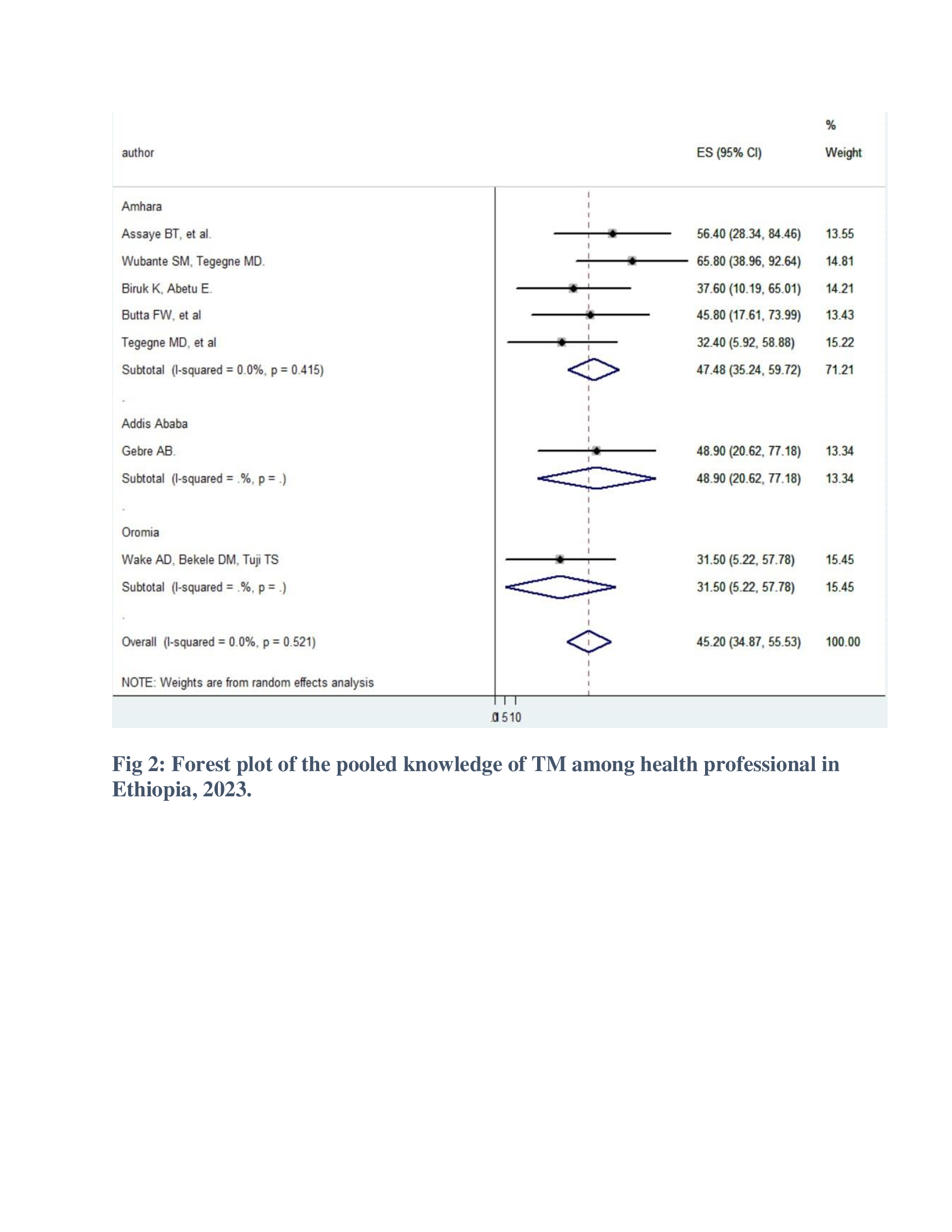

Supplement: S2 Fig — (TIFF) [file pone.0301044.s003.tiff]

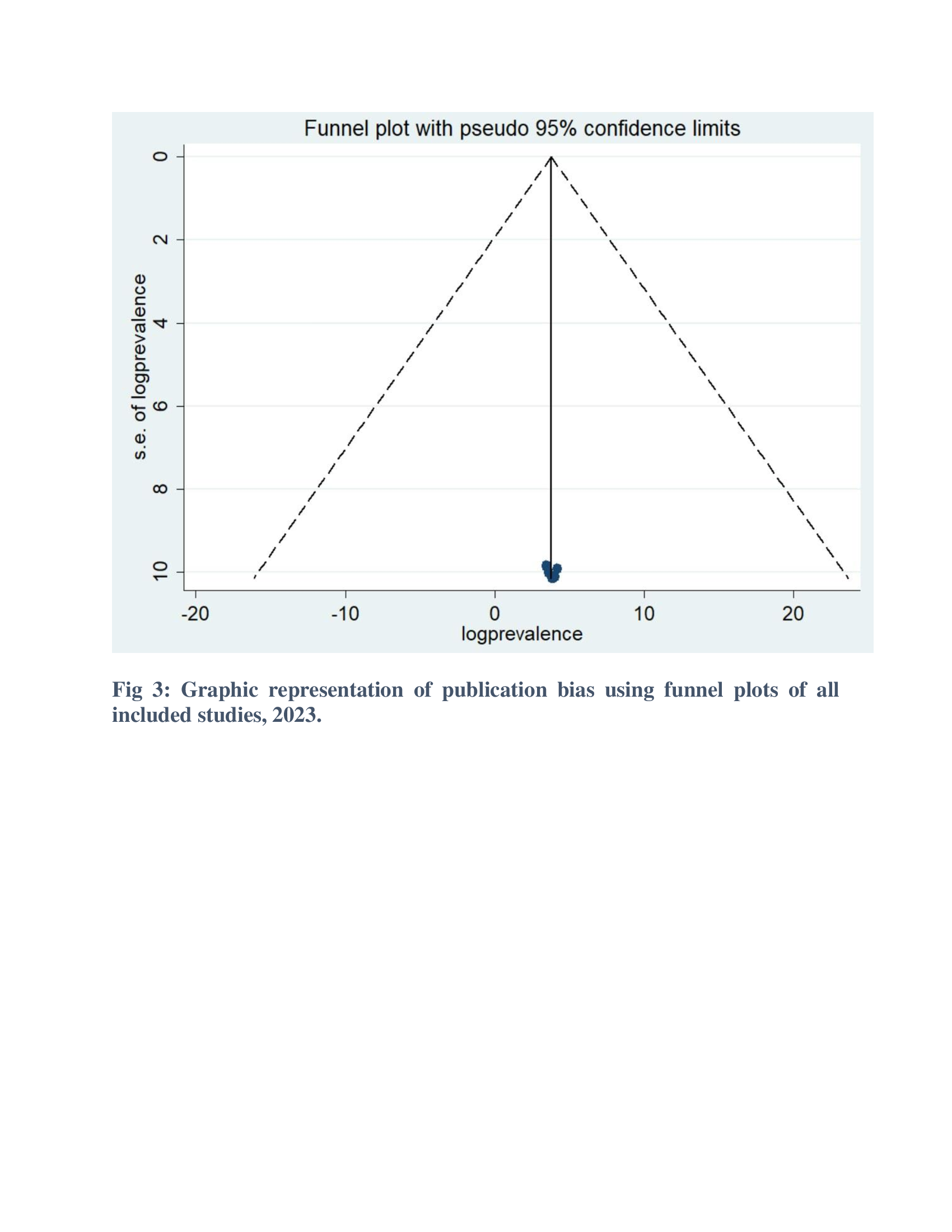

Supplement: S3 Fig — (TIFF) [file pone.0301044.s004.tiff]

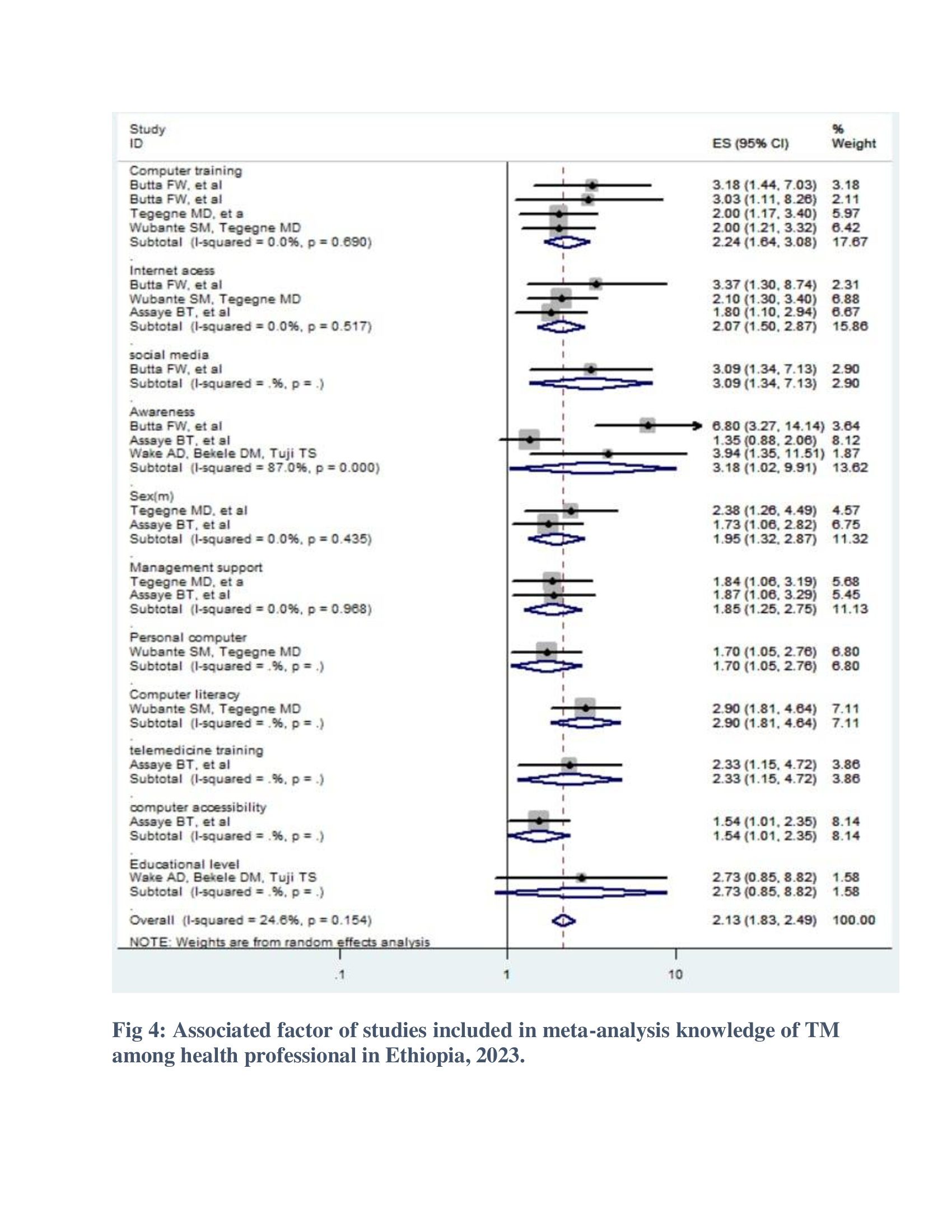

Supplement: S4 Fig — (TIFF) [file pone.0301044.s005.tiff]
