# Supplementary material for: Knowledge of telemedicine and its associated factors among health professional in Ethiopia: A systematic review and meta-analysis
Source: PLoS One. 2024 Apr 18;19(4):e0301044. doi: 10.1371/journal.pone.0301044 (PMC11025815; doi:10.1371/journal.pone.0301044)
Supplement: S1 Table — (DOCX) [file pone.0301044.s006.docx]

**S1 Table: Quality assessment Knowledge of telemedicine and its associated factors among health professional in Ethiopia: A systematic review and meta-analysis**.

| Author, year of  Study | Q1 | Q2 | Q3 | Q4 | Q5 | Q6 | Q7 | Q8 | Q9 | Total score (9%) |
| --- | --- | --- | --- | --- | --- | --- | --- | --- | --- | --- |
| Assaye BT, et al. 2021. | Y | Y | Y | Y | Y | Y | NA | Y | Y | 8 |
| Wubante SM, Tegegne MD. 2021 | Y | Y | Y | Y | Y | NA | Y | NA | Y | 7 |
| Biruk K, Abetu E.  2018 | Y | Y | Y | Y | Y | Y | Y | Y | Y | 9 |
| Gebre AB. 2021. | Y | Y | Y | Y | Y | Y | Y | Y | Y | 9 |
| Butta FW, et al. 2023 | Y | Y | Y | NA | Y | Y | Y | Y | Y | 8 |
| Tegegne MD, et al. 2023 | Y | Y | Y | Y | Y | Y | Y | Y | Y | 9 |
| Wake AD, Bekele DM, Tuji TS. 2020 | Y | Y | Y | Y | Y | Y | Y | Y | Y | 9 |
