# Supplementary material for: Knowledge of telemedicine and its associated factors among health professional in Ethiopia: A systematic review and meta-analysis
Source: PLoS One. 2024 Apr 18;19(4):e0301044. doi: 10.1371/journal.pone.0301044 (PMC11025815; doi:10.1371/journal.pone.0301044)
Supplement: S2 Table — (DOCX) [file pone.0301044.s007.docx]

**S2 Table: Descriptive summary of primary studies included in the meta-analysis knowledge of TM and associated factors among health professionals, 2023.**

| First author,  publication year | Region | Study area | Study design | Study population | Sampling  Technique | Data collection  Method | Sample  Size | R-rate |
| --- | --- | --- | --- | --- | --- | --- | --- | --- |
| Assaye BT, et al [20] 2021. | Amhara | Amhara Region Referral Hospitals | IBCS | HP | stratified | self-administered | 411 | 97.2% |
| Wubante SM, Tegegne MD. [17]2021 | Amhara | private hospitals | IBCS | HP | simple random | self-administered | 410 | 96.9% |
| Biruk K, Abetu E. [21]  2018 | Amhara | hospitals of North Gondar Administrative Zone | IBCS | HP | Proportionate stratified simple random | self-administered | 312 | 95.5% |
| Gebre AB. [22]2021. | AA | Addis Ababa Black loin hospital | FBCS mixed | HP | stratified random | A self-administered | 475 | 91% |
| Butta FW, et al [23] 2023 | Amhara | specialized teaching  referral hospital | IBCS | HP | simple random | A self-administered | 415 | 98.5% |
| Tegegne MD, et al [24] 2023 | Amhara | University of Gondar College | IBCS | Medical students | simple random | self-administered | 352 | 93.6% |
| Wake AD, Bekele DM, Tuji TS[25] 2020 | Oromia | four public hospitals of Arsi Zone | IBCS | hypertensive patients | simple random | interviewer-administered | 400 | 97.6% |
