# Supplementary material for: Knowledge of telemedicine and its associated factors among health professional in Ethiopia: A systematic review and meta-analysis
Source: PLoS One. 2024 Apr 18;19(4):e0301044. doi: 10.1371/journal.pone.0301044 (PMC11025815; doi:10.1371/journal.pone.0301044)
Supplement: Attachment — Submitted filename: PONE-D-23-43594.docx [file pone.0301044.s008.docx]

The manuscript examines a significant issue: one of the key barriers to the use of telehealth is doctors' lack of knowledge. While the structure and writing are appropriate, further investigation is required for some cases, as explained below.

- Two different abstracts are presented, which differ in their results section.
- It is recommended to add Telehealth to the keywords.
- The introduction requires significant editing.
- It is recommended to focus on the widespread use of telemedicine in various fields, especially in countries with limited resources, in the second paragraph. If desired, use the following reference. Hayavi-Haghighi MH, Alipour J. Applications, opportunities, and challenges in using Telehealth for burn injury management: A systematic review. Burns. 2023 Sep;49(6):1237-1248. doi: 10.1016/j.burns.2023.07.001. Epub 2023 Jul 13. PMID: 37537108.
- The second paragraph of the introduction presents the findings of the current study, therefore it is more appropriate for the discussion section. The last paragraph of the introduction, especially after stating the study's objective, contains information that is not suitable for this section and is somewhat appropriate for the conclusion or recommendations section.
- The process of scoring based on NOS and the number of questions will be explained.
- It is worth noting that the accuracy of the results may be impacted by the limited number of selected papers. It would be beneficial for the author to provide an explanation for this and address any corresponding shortcomings.
- It appears that there may have been some issues with the search process. It is worth noting that if Ethiopia is a key search term, as stated in the method, studies conducted outside of the country should not have been included in the initial search.
