# Supplementary material for: Knowledge of telemedicine and its associated factors among health professional in Ethiopia: A systematic review and meta-analysis
Source: PLoS One. 2024 Apr 18;19(4):e0301044. doi: 10.1371/journal.pone.0301044 (PMC11025815; doi:10.1371/journal.pone.0301044)
Supplement: Attachment — Submitted filename: point by point responce.docx [file pone.0301044.s009.docx]

**Date:** February 13, 2023

**Dear editorial board member (s) of** **PLOS ONE journal**

We have been recalled to revise the manuscript entitled **“Knowledge of telemedicine and its associated factors among health professional in Ethiopia: a systematic review and Meta-analysis”*,*** and identified by unique submission and revised **Manuscript Number: PONE-D-23-43594**  under **PLOS ONE** journal, which was submitted for publication. So, we received the editor(s)’ comments for the betterment of the manuscript before its publication.

Thank you editor for your comments, suggestion, directions, and questions. We are very happy in receiving constructive and invaluable comments for the betterment of the manuscript. Accordingly, we have considered all the comments, questions, directions, and suggestions and provided a point-by-point response letter.

Finally, we have submitted all the required documents in their revised form. We hope that we have addressed all the suggestions, directions, and raised questions and if you believe that point(s) is not addressed, please let us know.

**Thank you very much all editor (s)**

**On the behalf of the authors**

**Yours sincerely,**

**Correspondence author**

**Point-by-point response letter**

**Editor comments**

**Editor** the following changes must be made to your manuscript before moving on to the next stage.

1. Please ensure that your manuscript meets PLOS ONE's style requirements, including those for file naming. The PLOS ONE style templates can be found at.

**Author’s Response:** Thank you so much. Now it is corrected as plos one’s guideline .

2. We note that your Data Availability Statement is currently as follows: [there is no copy of data in this manuscript it original work

**Author’s Response:** Thank you so much. We have attached the data.

3. Please amend either the abstract on the online submission form (via Edit Submission) or the abstract in the manuscript so that they are identical.

**Author’s Response:** Thank you so much. We have update the abstract.

**Review Comments to the Author**

Please use the space provided to explain your answers to the questions above. You may also include additional comments for the author, including concerns about dual publication, research ethics, or publication ethics. (Please upload your review as an attachment if it exceeds 20,000 characters)

**Author’s Response:** Thank you so much. No concern of dual publication.

**Reviewer #1:** The manuscript examines a significant issue: one of the key barriers to the use of telehealth is doctors' lack of knowledge. While the structure and writing are appropriate, further investigation is required for some cases, as explained below.

• Two different abstracts are presented, which differ in their results section.

**Author’s Response:** Thank you so much. We have update the abstract.

• It is recommended to add Telehealth to the keywords.

**Author’s Response:** Thank you so much. We have added Telehealth to the keywords.

•The introduction requires significant editing.

**Author’s Response:** Thank you so much. We have modified the introduction.

• It is recommended to focus on the widespread use of telemedicine in various fields, especially in countries with limited resources, in the second paragraph. If desired, use the following reference. Hayavi-Haghighi MH, Alipour J. Applications, opportunities, and challenges in using Telehealth for burn injury management: A systematic review. Burns. 2023 Sep; 49(6):1237-1248. doi: 10.1016/j.burns.2023.07.001. Epub 2023 Jul 13. PMID: 37537108.

**Author’s Response:** Thank you so much. We have modified the introduction based on your recommendation article.

• The process of scoring based on NOS and the number of questions will be explained.
• It is worth noting that the accuracy of the results may be impacted by the limited number of selected papers. It would be beneficial for the author to provide an explanation for this and address any corresponding shortcomings.

**Author’s Response:** Thank you so much. We have NOS number of questions the score has be explained in the document.

• It appears that there may have been some issues with the search process. It is worth noting that if Ethiopia is a key search term, as stated in the method, studies conducted outside of the country should not have been included in the initial search.

**Author’s Response:** Thank you so much. We have remove Ethiopia in the keyword.

**Reviewer #2:** The authors have done a great job. A meta-analysis of studies from different regions of Ethiopia provides a summary of the state of telemedicine in the country. Understanding this will be useful for medical and educational institutions, government and health professionals.

**Author’s Response:** Thank you so much, for your suggestion

However, there are some comments that need to be corrected: line 95 "TM" is repeated. There is no need to include "TM" twice into the query. Line 177 and 178 - word "Awareness" should it started from low case letter ("awareness")? Line 196-197 please give some examples of % numbers in other countries. The quality of the Figures 1-4 should be improved! Figure 1. And Figure 3. Notes under the figures are unreadable Figure 2. And Figure 4. - Difficult or impossible to read words and digits.

**Author’s Response:**

1. Line 95 "TM" is repeated.

**Author’s Response:** Thank you so much. Now TM has removed.

2. There is no need to include "TM" twice into the query.

**Author’s Response:** Thank you so much. Now is used once.

3. Line 177 and 178 - word "Awareness" should it started from low case letter ("awareness")?

**Author’s Response:** Thank you so much. Now corrected as you asked.

4. Line 196-197 please give some examples of % numbers in other countries.

**Author’s Response:** Thank you so much. Now corrected as you asked, and the % of each country is written.

5. The quality of the Figures 1-4 should be improved! Figure 1. And Figure 3. Notes under the figures are unreadable Figure 2. And Figure 4. - Difficult or impossible to read words and digits.
6. **Author’s Response:** Thank you so much. Now all the figure 1-figure 4 are improved we have analyzed again and the figure is improved.
1. Thank you for stating the following in your Competing Interests section:

**Author’s Response:** We include the Competing Interests in this cover letter please change the online submission form on behalf me.

**With regards!**

**Thank you!**

**Yours sincerely**

**Correspondence author**
